# PIK3CA-Related Overgrowth Syndrome (PROS) and Angiosarcoma: A Case Report

**Published:** 2020-04-17

**Authors:** Brielle Weinstein, Evita Henderson-Jackson, C. Wayne Cruse, Andrew S. Brohl

**Affiliations:** ^a^Department of Plastic Surgery, University of South Florida, Tampa; ^b^Departments of Pathology; ^c^Cutaneous Oncology; ^d^Sarcoma, H. Lee Moffitt Cancer Center and Research Institute, Tampa, Fla

**Keywords:** PIK3CA-related overgrowth syndrome, angiosarcoma, CLOVES, cutaneous oncology, cancer genetics

## DESCRIPTION

A 73-year-old man presented with cutaneous angiosarcoma of the left temporal scalp. In addition, he had a constellation of clinical findings including hemihypertrophy, lipomatous overgrowth with truncal distribution, scoliosis, epidermal nevi, and later, with tissue identification of *PIK3CA* mutation, he was diagnosed with PIK3CA-related overgrowth syndrome (PROS), likely CLOVES syndrome.

## QUESTIONS

1. What is CLOVES syndrome?
2. What is PIK3CA Related Overgrowth Syndrome (PROS) and which pathways can this gene affect?
3. Describe the clinical presentations of cutaneous angiosarcoma.
4. What are appropriate comprehensive oncologic treatment regimens for cutaneous angiosarcoma?

## DISCUSSION

In recent years with the progression of genetic research, there has been a paradigm shift in both oncology and congenital research toward a focus on targeted genetic diagnoses and subsequent treatments. We present a case of a patient with PROS, likely CLOVES syndrome, and angiosarcoma, 2 distinct pathologies that share a common genetic pathway. We report, to our knowledge, the first case of angiosarcoma in a patient with PROS. Although a single case, we hypothesize that both conditions might share a common pathophysiology. For one, several of the clinical manifestations of CLOVES (hypertrophy, lymphatic overgrowth, vascular malformations) may be at risk for degeneration to angiosarcoma. Second, the somatic *PIK3CA* mutation that causes CLOVES might be a “first-hit” genetic driver of the subsequent angiosarcoma. It is notable that the *PIK3CA* mutation in this case was identified at very high allele frequency within the tumor specimen, suggestive of a second-hit structural event leading to at least a partial loss of heterozygosity. Given the extreme rarity of both of these diseases, even if CLOVES or PROS predisposes for angiosarcoma, we expect cases to be rare. This case therefore adds to literature of this potential interesting association that might lead to further insight into the pathophysiology of an uncommon, aggressive malignancy. Additional reports would be needed to confirm the relationship of these 2 conditions.

Congenital lipomatous asymmetric overgrowth of the trunk with lymphatic, capillary, venous, and combined type vascular malformations, epidermal nevi, scoliosis, and spinal abnormalities (CLOVES) is a clinical syndrome now known to be caused by mosaic somatic activating mutations in *PIK3CA.*^1^^,^^2^ CLOVES is one of a spectrum of overgrowth phenotypes that may result from *PIK3CA* mutation, now falling under the umbrella term “*PIK3CA*-related overgrowth spectrum” (PROS).^3^ Other PROS conditions include macrodactyly, fibroadipose hyperplasia or overgrowth, hemihyperplasia multiple lipomatosis, and several megaencephaly conditions. The PI3K-AKT-mTOR signaling pathway is one of the most frequently dysregulated pathways in cancer. Gain-of-function *PIK3CA* mutations are seen in almost every tumor type and affect approximately 13% of cancers overall.^4^ Not surprisingly, patients affected by CLOVES are likely to have at least a modestly increased risk of development of malignancy. Most notably, Wilms tumor has been observed relatively frequently (3.3%) in the CLOVES population compared with the general population (˜1/10,000).^5^ An association of CLOVES with other malignancies, however, has not been well established.

Angiosarcoma is a rare soft-tissue sarcoma of endothelial origin. The PI3K-AKT-mTOR pathway is significantly overactivated in this tumor type, though *PIK3CA* mutations specifically are uncommon.^6^ Angiosarcomas can also arise in the setting of lymphedema (ie, Stewart-Treves syndrome)^7^ and rarely can result from degeneration of congenital vascular malformation.^8^ Similar to our patient, many cutaneous angiosarcomas can begin as a purpuric lesion with pigmentation that later expanding to a large patch covering much of the local area.

**Figure 1:**
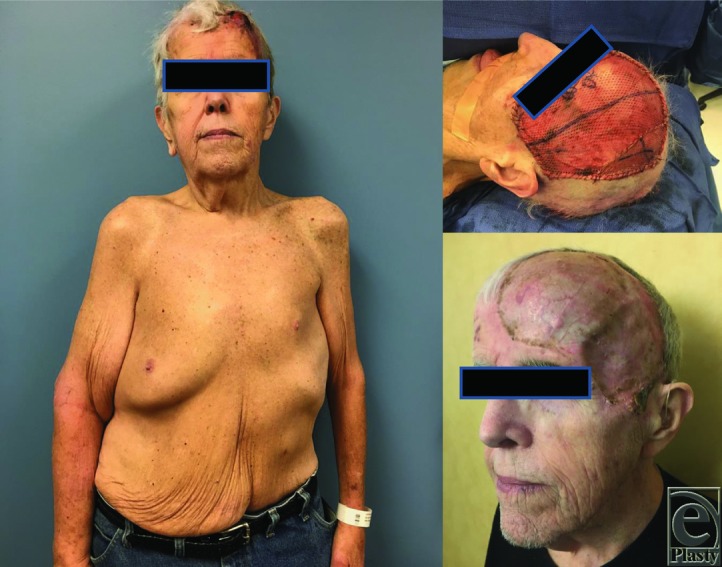
(*a*-*c*) Clinical photographs of the patient demonstrating (*a*) hemihypertrophy with truncal distribution and tumor of the left scalp. (*b*) Left scalp defect following resection through skin, subcutaneous tissue, fascia, muscle, and galea with split-thickness skin graft and (*c*) 2 weeks after operation with a well-healed graft.

**Figure 2:**
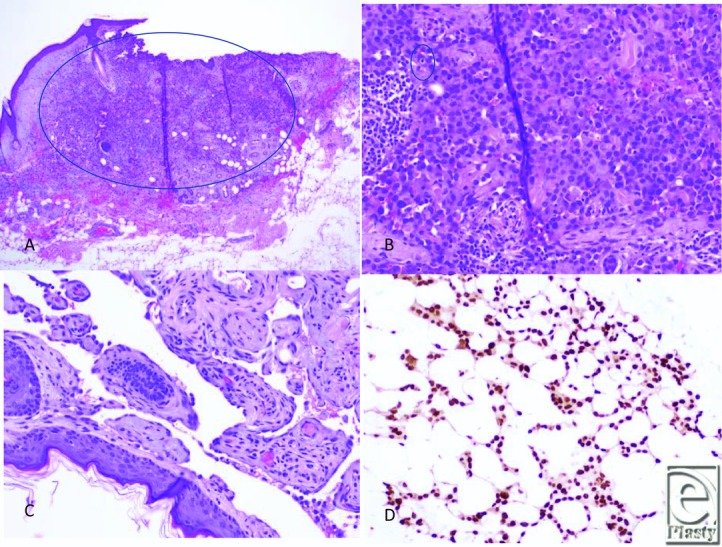
Histologic images of cutaneous angiosarcoma. (*a*) Left scalp excision revealed multiple foci of malignant cells involving the dermis and superficially involving subcutaneous soft tissue. Note this area demonstrates a solid area of growth of epithelioid cells. (H&E, ×4). (*b*) Areas of the tumor reveal malignant epithelioid cells with vague rudimentary vasoformation. The epithelioid cells exhibit round/ovoid irregular nuclei with abundant cytoplasm. Mitotic figure is noted (H&E, ×20). (*c*) Other areas of the tumor demonstrate anastomosing vessels lined by plump, hyperchromatic endothelial cells with focal papillations (H&E, ×20). (*d*) The tumor shows vasoformative growth within subcutaneous tissue. Tumor cells are positive for ERG expression (Immunostain, ×20). HE indicates hematoxylin and eosin.

Angiosarcoma can be an aggressive tumor and patients may meet with a medical oncologist for neoadjuvant cytotoxic chemotherapy, a radiation oncologist for postoperative radiation, as well as a plastic surgeon for wide local excision and reconstruction prior to treatment. Our patient began treatment with neoadjuvant paclitaxel, completing 3 cycles with excellent clinical response. At this time, he underwent excision of left scalp angiosarcoma including skin, fat muscle, and galea with split-thickness skin graft reconstruction (Fig 1). Final pathology confirmed angiosarcoma, including positive immunostaining for ERG, a highly sensitive and specific marker for this disease (Fig 2).^9^ *PIK3CA* mutational testing was performed on the tumor specimen, given the patient's constellation of findings and confirmed p.E545D mutation with an allele frequency of 79.8%. Adjuvant radiation therapy was completed postoperatively to a dose of 60 Gy in 2-Gy fractions. He remains well and without any evidence of disease at 2 months of postoperative follow-up.
